# Decreased Circulating C3 Levels and Mesangial C3 Deposition Predict Renal Outcome in Patients with IgA Nephropathy

**DOI:** 10.1371/journal.pone.0040495

**Published:** 2012-07-06

**Authors:** Seung Jun Kim, Hyang Mo Koo, Beom Jin Lim, Hyung Jung Oh, Dong Eun Yoo, Dong Ho Shin, Mi Jung Lee, Fa Mee Doh, Jung Tak Park, Tae-Hyun Yoo, Shin-Wook Kang, Kyu Hun Choi, Hyeon Joo Jeong, Seung Hyeok Han

**Affiliations:** 1 Department of Internal Medicine, Yonsei University College of Medicine, Seoul, Korea; 2 Department of Pathology, Yonsei University College of Medicine, Seoul, Korea; 3 Severance Biomedical Science Institute, Brain Korea 21, Yonsei University, Seoul, Korea; Institut national de la santé et de la recherche médicale (INSERM), France

## Abstract

**Background and Aims:**

Mesangial C3 deposition is frequently observed in patients with IgA nephropathy (IgAN). However, the role of complement in the pathogenesis or progression of IgAN is uncertain. In this observational cohort study, we aimed to identify the clinical implications of circulating C3 levels and mesangial C3 deposition and to investigate their utility as predictors of renal outcomes in patients with IgAN.

**Methods:**

A total of 343 patients with biopsy-proven IgAN were enrolled between January 2000 and December 2008. Decreased serum C3 level (hypoC3) was defined as C3 <90 mg/dl. The study endpoint was end-stage renal disease (ESRD) and a doubling of the baseline serum creatinine (D-SCr).

**Results:**

Of the patients, there were 66 patients (19.2%) with hypoC3. During a mean follow-up of 53.7 months, ESRD occurred in 5 patients (7.6%) with hypoC3 compared with 9 patients (3.2%) with normal C3 levels (P = 0.11). However, 12 patients (18.2%) with hypoC3 reached D-SCr compared with 17 patients (6.1%) with normal C3 levels [Hazard ratio (HR), 3.59; 95% confidence interval (CI), 1.33–10.36; P = 0.018]. In a multivariable model in which serum C3 levels were treated as a continuous variable, hypoC3 significantly predicted renal outcome of D-SCr (per 1 mg/dl increase of C3; HR, 0.95; 95% CI, 0.92–0.99; P = 0.011). The risk of reaching renal outcome was significantly higher in patients with mesangial C3 deposition 2+ to 3+ than in patients without deposition (HR 9.37; 95% CI, 1.10–80.26; P = 0.04).

**Conclusions:**

This study showed that hypoC3 and mesangial C3 deposition were independent risk factors for progression, suggesting that complement activation may play a pathogenic role in patients with IgAN.

## Introduction

IgA nephropathy (IgAN) is most common primary glomerulonephritis worldwide [1]. Patients with IgAN have a variable clinical course, ranging from a totally benign condition to progressive deterioration in kidney function over time. Approximately 20 to 30% of the patients with IgAN will eventually develop end stage renal disease (ESRD) within 20 to 25 years after disease onset [2]. Previous studies have identified clinical and pathologic features associated with adverse outcomes. These include heavy proteinuria, reduced renal function, hypertension at the time of diagnosis, interstitial fibrosis, and glomerular sclerosis [3]–[5]. However, there are no available serologic tests that can be employed to assess disease activity or to predict renal outcomes in these patients.

Although IgA deposits within the mesangium are a key diagnostic finding in IgAN, mesangial C3 deposition is also frequently observed. However, the role of complement activation in the pathogenesis or progression of IgAN is uncertain [6]. In previous studies, dimeric and polymeric IgA have been found to activate complement system in the glomeruli via the alternative or lectin pathway, thus leading to glomerular damage [7]–[12]. It was also reported that systemic complement activation occurs in patients with IgAN [13], [14]. Specifically, Zwirner showed that activated C3 was associated with increased proteinuria and subsequent deterioration in kidney function in these patients, suggesting that systemic complement activation might play a role in renal injury in this glomerulopathy [14]. However, their findings have not yet been validated, thus whether hypocomplementemia may have prognostic value for predicting renal outcomes is currently unknown. Therefore, we undertook an observational cohort study to determine the clinical implications of decreased serum C3 levels (hypoC3) and to investigate its utility as a predictor of renal outcomes in patients with IgAN. We also examined clinical features and outcomes according to the pathologic findings, particularly mesangial C3 deposition in these patients.

**Figure 1:**
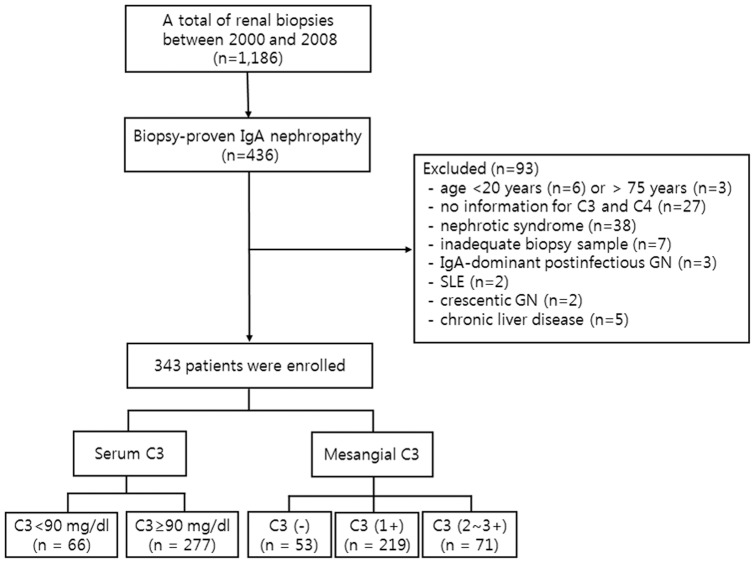
Flow diagram of the study. IgA nephropathy was diagnosed in 436 patients between January 2000 and December 2008. Excluding 93 patients, a total of 343 patients were enrolled. eGFR, estimated glomerular filtration rate; GN, glomerulonephritis; SLE, systemic lupus erythematosus.

## Methods

### Ethics statement

The study was carried out in accordance with the Declaration of Helsinki and approved by the Institutional Review Board of Yonsei University Health System Clinical Trial Center. We obtained informed written consent from all participants involved in our study.

**Figure 2:**
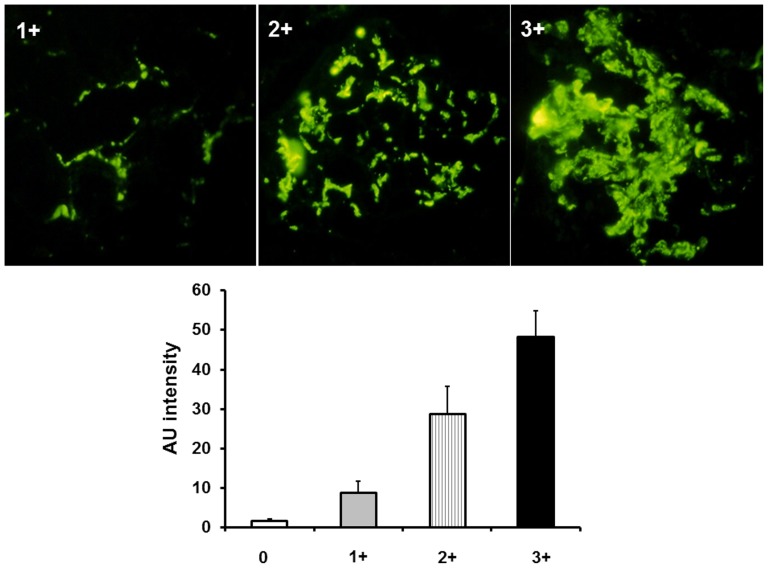
Representative pictures of immunofluorescence staining of mesangial C3 1+ to 3+. Immunofluorescence intensity was quantified by ImageJ software.

### Patients

Renal biopsy was performed in 1181 patients at Yonsei University Severance Hospital between January 2000 and December 2008. Among these patients, 436 were diagnosed with IgAN. Patients with Henoch-Schonlein purpura were considered ineligible. Our routine practice to assess glomerular disease encompasses the measurement of serum concentrations of complement. However, patients in whom serum complement levels were not available at the time of renal biopsy were excluded (n = 27). We also excluded patients who had features of IgA-dominant acute post-infectious glomerulonephritis exhibiting hypocomplementemia, diffuse glomerular endocapillary hypercellularity, and subepithelial humps on electron microscopy (n = 3) [15], and patients who had features of lupus nephritis, such as the presence of typical autoantibodies and “full house” immunofluorescence pattern which was defined as the mesangial co-deposits of IgG, IgA, IgM, and/or C1q (n = 2) [16]. In addition, patients with age <20 years (n = 6) or >75 years (n = 3), inadequate biopsy sample with the number of glomeruli ≤7 (n = 7), and patients who initially presented with nephrotic syndrome (n = 38), crescentic glomerulonephritis (n = 2), and advanced chronic liver diseases (n = 5) were also excluded. Therefore, a total of 343 patients were included in this study (Figure 1).

**Figure 3:**
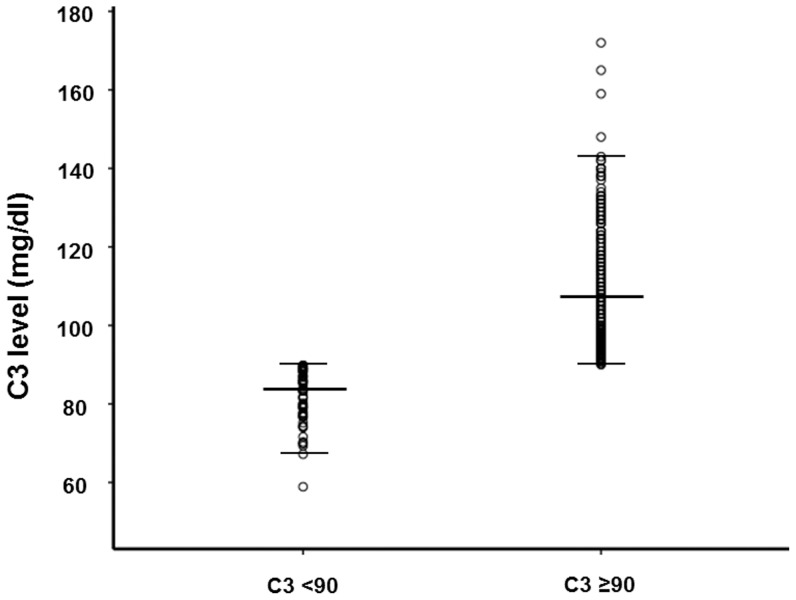
A scattered plot of each level of serum C3 between patients with C3 levels <90 mg/dl and patients with C3 levels ≥90 mg/dl.

**Table 1:** Demographic, clinical and biochemical characteristics.

	All (n = 343)	C3 <90 mg/dl (n = 66)	C3 ≥90 mg/dl (n = 277)	P-value
Age (years)	34.5±11.7	31.1±9.2	35.4±12.1	0.002
Male gender (n, %)	159 (46.4%)	30 (45.5%)	129 (46.6%)	0.87
Hypertension (n, %)	85 (24.8%)	8 (12.1%)	77 (27.8%)	0.008
Diabetes (n, %)	4 (1.2%)	1 (1.5%)	3 (1.1%)	0.77
Coronary artery disease (n, %)	8 (2.3%)	2 (3.0%)	6 (2.2%)	0.68
Hepatitis B antigen positivity (n, %)	15 (4.4%)	2 (3.2%)	13 (5.1%)	0.74
Hepatitis C antibody positivity (n, %)	1 (0.3%)	0 (0%)	1 (0.6%)	1.00
Episodes of gross hematuria (n, %)	93 (27.1%)	16 (24.2%)	77 (27.8%)	0.56
Body mass index (kg/m^2^)	22.7±3.3	20.9±2.4	23.2±3.3	<0.001
Systolic blood pressure (mmHg)	125.7±15.3	121.5±11.1	126.8±16.0	0.002
141 to 160 mmHg (n, %)	34 (9.9%)	7 (10.6%)	27 (9.7%)	
161 to 180 mmHg (n, %)	2 (0.6%)	0 (0%)	2 (0.7%)	
>180 mmHg (n, %)	2 (0.6%)	0 (0%)	2 (0.7%)	
MAP (mmHg)	94.2±11.4	91.4±9.5	94.8±11.7	0.031
Blood urea nitrogen (mg/dl)	15.0±5.6	15.2±6.4	15.0±5.5	0.74
Creatinine (mg/dl)	1.1±0.4	1.1±0.4	1.1±0.4	0.34
eGFR (ml/min/1.73 m^2^)	79.8±24.2	78.8±26.1	80.0±23.8	0.71
eGFR <30 (n, %)	9 (2.6%)	3 (4.5%)	6 (2.2%)	
30≤ eGFR <60 (n, %)	65 (19.0%)	15 (22.7%)	50 (18.1%)	
eGFR ≥60 (n, %)	269 (78.4%)	48 (72.7%)	221 (79.8%)	
Protein (g/dl)	6.8±0.6	6.6±0.6	6.8±0.6	0.10
Albumin (g/dl)	4.1±0.5	4.0±0.5	4.1±0.5	0.10
Uric acid (mg/dl)	5.7±1.6	5.6±1.7	5.8±1.6	0.45
Cholesterol (mg/dl)	181.8±38.5	170.8±39.9	184.4±37.8	0.01
Triglycerides (mg/dl)	132.3±91.0	106.1±50.1	139.2±97.9	0.002
Urine PCR (mg/mg)	1.3±1.4	1.2±1.4	1.4±1.4	0.34
IgG (mg/dl)	1238.7±301.2	1200.7±294.2	1247.9±302.9	0.30
IgA (mg/dl)	309.5±103.8	296.5±95.8	312.7±105.7	0.28
C3 (mg/dl)	104.2±17.1	82.0±6.8	109.5±14.3	<0.001
C-reactive protein (mg/l)*	1.0 (0.3–2.72)	1.0 (0.5–1.3)	1.1 (0.5–3.1)	0.53
Medications				
ACE inhibitors or ARBs	215 (62.7%)	39 (59.1%)	176 (63.5%)	0.50
Diuretics	60 (17.5%)	7 (10.6%)	53 (19.1%)	0.09
Other antihypertensive drugs	53 (15.5%)	6 (9.1%)	47 (17.0%)	0.11
More than 2 antihypertensive drugs	50 (14.6%)	6 (9.1%)	44 (15.9%)	0.16
More than 3 antihypertensive drugs	18 (5.2%)	1 (1.5%)	17 (6.1%)	0.22
HMG-CoA reductase inhibitors	49 (14.3%)	6 (9.1%)	43 (15.5%)	0.18
Corticosteroid	10 (2.9%)	2 (3.0%)	8 (2.9%)	0.95
Cyclosporine	3 (0.9%)	0 (0%)	3 (1.1%)	0.40
Time to renal biopsy (months)*	12 (3–36)	11 (3–30)	12 (3–36)	0.48

Data are presented as n (%) or mean ± SD or *median and interquartile range.

MAP, mean arterial pressure; eGFR, estimated glomerular filtration rate; PCR, protein to creatinine ratio; Ig, immunoglobulin; C, complement; ACE, angiotensin converting enzyme; ARB, angiotensin II receptor blocker.

### Data collection

At the time of the renal biopsy, patients' demographic and clinical data such as age, gender, blood pressure, episode of gross hematuria, and presence of hypertension were recorded. Hypertension was defined as systolic blood pressure >140 mmHg or 90 mmHg and the need for antihypertensive medication to maintain pressures below these levels. In addition, laboratory parameters such as serum albumin, blood urea nitrogen, creatinine, uric acid, total cholesterol, triglycerides, C-reactive protein (CRP) and urinary protein-to-creatinine ratio (UPCR) were measured. Serum concentrations of immunoglobulins (Igs) A or G and C3 were measured by immunoturbidimetry (Cobas C501, Roche, Mannhein, Germany). Using this method, the normal reference range of C3 levels are 90–180 mg/dl. We calculated eGFR using the Modification of Diet in Renal Disease (MDRD) study equation [17].

**Figure 4:**
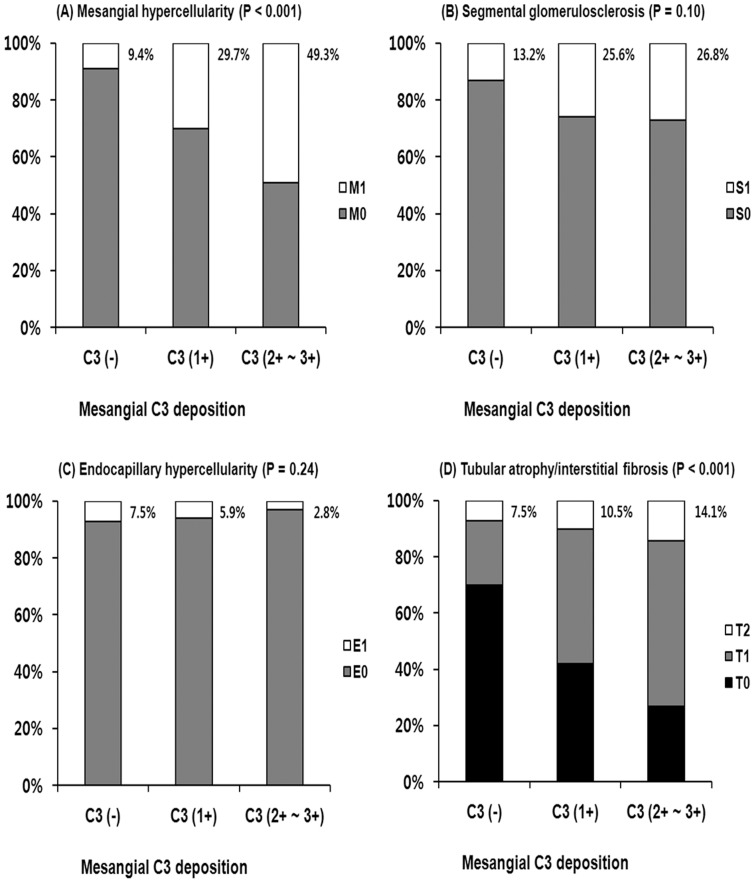
The histopathologic grades such as (A) mesangial hypercellularity, (B) segmental glomerulosclerosis, (C) endocapillary hypercellularity, and (D) tubular atrophy/interstitial fibrosis according to mesangial C3 deposition. Mesangial hypercellularity (C3 deposition 0, 9.4%; 1+, 29.7%; 2+∼3+, 49.3%; P<0.001) and high-grade tubular atrophy/interstitial fibrosis (C3 deposition 0, 7.5%; 1+, 10.5%; 2+∼3+, 14.1%; P<0.001) were more prominent as the mesangial area of C3 deposition increased.

**Figure 5:**
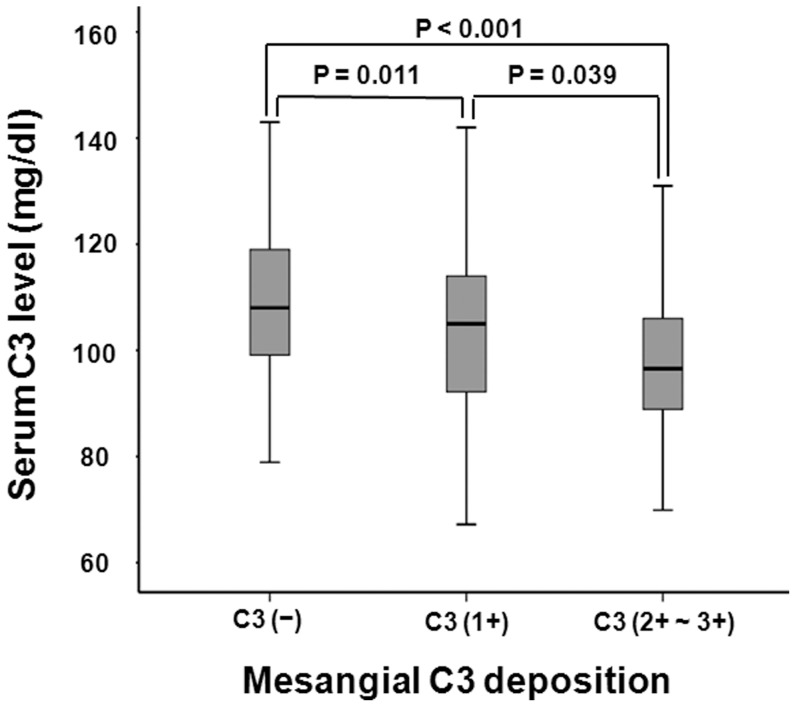
Comparison of serum C3 levels according to mesangial C3 deposition. Serum C3 levels decreased significantly from 0 to 2+∼3+ mesangial C3 deposition (0, 111.7±18.0; 1+, 104.3±17.1; 2+∼3+, 98.6±14.2 mg/dl; P<0.001).

**Table 2:** Comparison of histopathologic features from renal biopsies between patients with and without decreased C3 levels.

	All (n = 343)	C3 <90 mg/dl (n = 66)	C3 ≥90 mg/dl (n = 277)	P-value
Mesangial hypercellularity				0.81
≤50% of the glomeruli (M0)	238 (69.4%)	45 (68.2%)	193 (69.7%)	
>50% of the glomeruli (M1)	105 (30.6%)	21 (31.8%)	84 (30.3%)	
Segmental glomerulosclerosis				0.30
Absent (S0)	261 (76.1%)	47 (71.2%)	214 (77.3%)	
Present (S1)	82 (23.9%)	19 (28.8%)	63 (22.7%)	
Endocapillary hypercellularity				0.16
Absent (E0)	324 (94.5%)	60 (90.9%)	264 (95.3%)	
Present (E1)	19 (5.5%)	6 (9.1%)	13 (4.7%)	
Tubular atrophy/interstitial fibrosis				0.07
0–25% of cortical area (T0)	148 (43.1%)	23 (34.8%)	125 (45.1%)	
25–50% of cortical area (T1)	158 (46.1%)	31 (47.0%)	127 (45.8%)	
>50% of cortical area (T2)	37 (10.8%)	12 (18.2%)	25 (9.0%)	
Arteriosclerosis	70 (20.4%)	12 (18.2%)	58 (20.9%)	0.62
Mesangial IgG deposition (n, %)				0.45
Negative	204 (59.5%)	36 (54.5%)	168 (60.6%)	
1+	126 (36.7%)	26 (39.4%)	100 (36.1%)	
2+	11 (3.2%)	4 (6.1%)	7 (2.5%)	
3+	2 (0.6%)	0 (0.0%)	2 (0.7%)	
2+ and 3+	13 (3.8%)	4 (6.1%)	9 (3.2%)	
Mesangial IgA deposition (n, %)				0.77
1+	104 (30.3%)	21 (31.8%)	83 (30.0%)	
2+	168 (49.0%)	29 (43.9%)	139 (50.1%)	
3+	71 (20.7%)	16 (24.3%)	55 (19.9%)	
2+ and 3+	239 (69.7%)	45 (68.2%)	194 (70.0%)	
Mesangial C3 deposition (n, %)				0.011
Negative	53 (15.5%)	3 (4.5%)	50 (18.1%)	
1+	219 (63.8%)	44 (66.7%)	175 (63.2%)	
2+	63 (18.4%)	16 (24.3%)	47 (17.0%)	
3+	8 (2.3%)	3 (4.5%)	5 (1.8%)	
2+ and 3+	71 (20.7%)	19 (28.8%)	52 (18.8%)	

Data are presented as n (%).

Ig, immunoglobulin; C, complement.

### Renal biopsy

All renal biopsy specimens were re-assessed blindly by a single pathologist using the Oxford classification [18]. The biopsy specimens were processed for light microscopy, immunofluorescence study, and electron microscopy. IgAN was diagnosed by the following findings: (1) the presence of predominant IgA deposits (at least 1+) mainly in the mesangium by immunofluorescence, (2) the presence of mesangial electron-dense deposits by electron microscopic examination, and (3) the absence of other systemic inflammatory diseases such as systemic lupus erythematosus. The mesangial hypercellularity score was preferred to the percentage of glomeruli showing severe mesangial hypercellularity. The cutoff for the mesangial hypercellularity score was 0.5. Segmental glomerulosclerosis and endocapillary hypercellularity were categorized as either present or absent. Tubular atrophy/interstitial fibrosis was classified as T0 (0–25% of cortical area), T1 (26–50% of cortical area), or T2 (>50% of cortical area) [18]. We quantified the immunofluorescence staining of C3 deposition in the mesangial area by ImageJ software v1.60 (NIH, Bethesda, Maryland, USA; online at http://rsbweb.nih.gov/ij). With the use of this method, quantification of immunofluorescence was expressed as arbitrary unit (AU), which was calculated as (mean pixel intensity X glomerular area)/100,000. Mesangial C3 deposits were classified into four groups: 0, AU level <5; 1+, 5≤ AU level <20; 2+, 20≤ AU level <40; and 3+, 40≤ AU level (Figure 2).

**Figure 6:**
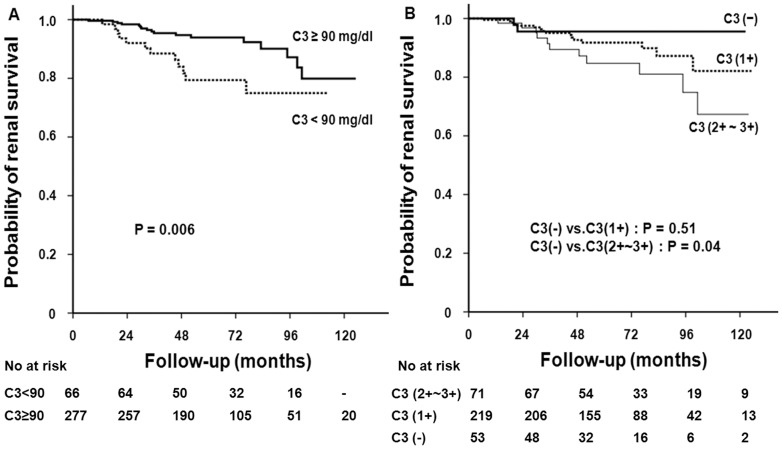
Kaplan-Meier analyses of cumulative renal survival of patients with IgA nephropathy based on (A) serum C3 level and (B) mesangial C3 deposition. (A) A 10-year renal survival rate was significantly lower in patients with C3 levels <90 mg/dl than those with C3 levels ≥90 mg/dl (P = 0.006). (B) A 10-year survival in patients with 2+ and 3+ mesangial deposition of C3 was lower than in those without C3 deposition (P = 0.04).

**Figure 7:**
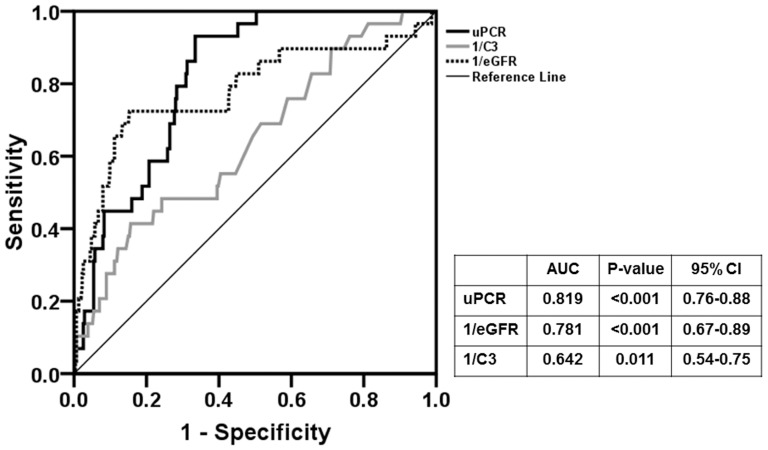
ROC curve analysis for renal outcome of the doubling of the baseline serum creatinine. Serum C3 levels had a significant predictive value for renal outcome (AUC = 0.642, P = 0.011), although the predictive value of serum C3 was lower than UPCR (AUC = 0.819, P<0.001) or eGFR (AUC = 0.781, P<0.001).

**Figure 8:**
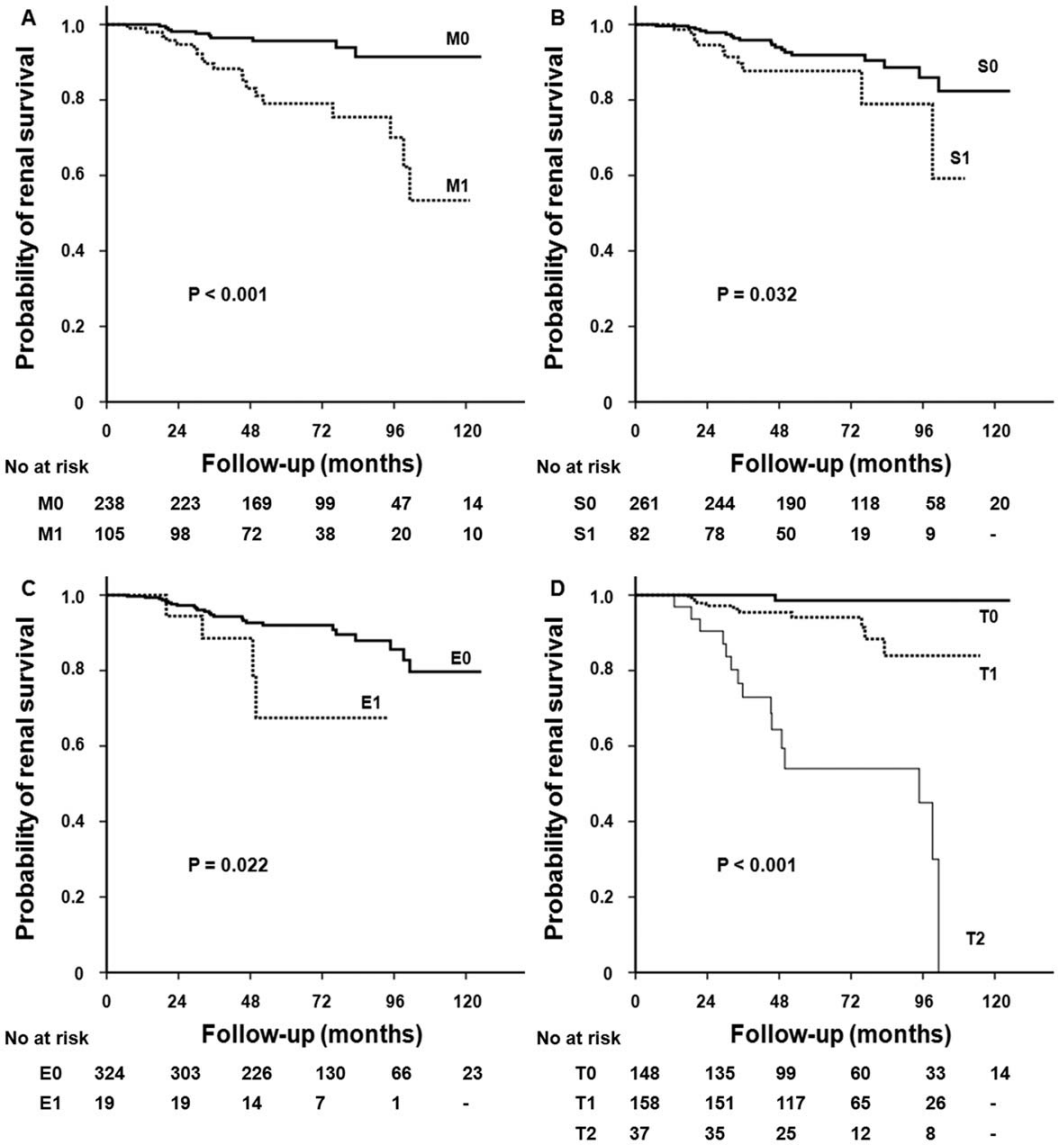
Kaplan-Meier analyses of cumulative renal survival of patients with IgA nephropathy according to histopathologic features including (A) mesangial hypercellularity, (B) segmental glomerulosclerosis, (C) endocapillary hypercellularity, and (D) tubular atrophy/interstitial fibrosis. Patients with mesangial hypercellularity, segmental glomerulosclerosis, endocapillary hypercellularity, and tubular atrophy/interstitial fibrosis had significantly lower renal survival than those without such findings (P<0.05).

**Table 3:** Incidence of patients with doubling of serum creatinine and ESRD according to decreased C3 levels and mesangial C3 deposition.

	Serum C3					Mesangial C3 deposition						
	C3 <90 mg/d		C3 ≥90 mg/dl		P-value	C3(−)		C3(+)		C3(2+∼3+)		P-value
	N (%)	/1000 patient -years	N (%)	/1000 patient -years		N (%)	/1000 patient -years	N (%)	/1000 patient -years	N (%)	/1000 patient -years	
ESRD	5 (7.6%)	15.67	9 (3.2%)	7.57	0.11	1 (1.9%)	5.05	9 (4.1%)	9.30	4 (5.6%)	11.73	0.30
Doubling of Scr	12 (18.2%)	34.48	17 (6.1%)	12.62	0.002	2 (3.8%)	5.05	16 (7.3%)	15.50	11 (15.5%)	29.33	0.016

ESRD, end-stage renal disease; C, complement; N, number; Scr, serum creatinine.

**Table 4:** Cox regression models for renal outcome of the doubling of the baseline serum creatinine.

	HR	95% CI	P-value	HR	95% CI	P-value
**Model 1**						
eGFR (per 1 ml/min/1.73 m^2^ increase)	0.96	0.93–0.98	<0.001	0.95	0.93–0.98	<0.001
Urinary PCR (per 1 mg/mg increase)	1.70	1.36–2.12	<0.001	1.52	1.26–1.82	<0.001
Serum C3 (per 1 mg/dl increase)	0.96	0.93–0.98	0.002	-	-	-
Patients with hypoC3 (vs. no)	-	-	-	3.59	1.56–8.28	0.003
**Model 2**						
eGFR (per 1 ml/min/1.73 m^2^ increase)	0.97	0.95–0.99	0.037	-	-	-
Urinary PCR (per 1 mg/mg increase)	1.63	1.27–2.08.	<0.001	-	-	-
Tubular atrophy/interstitial fibrosis				-	-	-
0–25% of cortical area (T0)	Reference					
25–50% of cortical area (T1)	7.632	0.91–63.6	0.061	-	-	-
>50% of cortical area (T2)	35.81	4.03–217.9	0.001	-	-	-
Mesangial C3 deposition						
0	Reference					
1+	6.03	0.93–39.00	0.059	-	-	-
2+∼3+	14.24	2.03–99.87	0.008	-	-	-
**Model 3**						
eGFR (per 1 ml/min/1.73 m^2^ increase)	0.98	0.95–0.99	0.038	0.97	0.95–0.99	0.031
Urinary PCR (per 1 mg/mg increase)	1.86	1.42–2.44	<0.001	1.71	1.32–2.21	<0.001
Tubular atrophy/interstitial fibrosis						
0–25% of cortical area (T0)	Reference			Reference		
25–50% of cortical area (T1)	8.20	0.97–69.4	0.053	8.50	1.01–71.43	0.049
>50% of cortical area (T2)	35.26	4.04–207.5	0.001	34.90	3.93–209.9	0.001
Mesangial C3 deposition						
0	Reference			Reference		
1+	3.75	0.63–22.3	0.147	5.64	0.80–39.55	0.082
2+∼3+	8.76	1.39–55.2	0.021	14.03	1.86–105.77	0.010
Serum C3 (per 1 mg/dl increase)	0.95	0.93–0.98	0.004	-	-	-
Patients with hypoC3 (vs. no)	-	-	-	3.35	1.41–7.99	0.006
**Model 4**						
eGFR (per 1 ml/min/1.73 m^2^ increase)	0.97	0.95–0.99	0.030	0.97	0.95–0.99	0.021
Urinary PCR (per 1 mg/mg increase)	1.78	1.29–2.46	<0.001	1.65	1.21–2.26	0.002
Tubular atrophy/interstitial fibrosis						
0–25% of cortical area (T0)		Reference			Reference	
25–50% of cortical area (T1)	8.14	0.94–70.59	0.057	8.09	0.94–69.6	0.057
>50% of cortical area (T2)	32.41	3.61–191.44	0.002	30.42	3.31–179.2	0.003
Mesangial C3 deposition						
0		Reference			Reference	
1+	4.58	0.62–33.70	0.135	7.25	0.69–58.89	0.064
2+∼3+	10.17	1.33–77.9	0.026	16.18	1.89–138.36	0.011
Serum C3 (per 1 mg/dl increase)	0.96	0.93–0.99	0.019	-	-	-
Patients with hypoC3 (vs. no)	-	-	-	2.73	1.09–6.86	0.032

Data are reported as hazard ratio (HR) and 95% confidence interval (CI).

Model 1: age, sex, presence of gross hematuria, mean arterial blood pressure, eGFR, proteinuria, treatment, and serum C3 levels.

Model 2: age, sex, presence of gross hematuria, mean arterial blood pressure, eGFR, proteinuria, treatment, and pathologic findings.

Model 3: Model 2+serum C3 levels.

Model 4: Model 3+BMI, total cholesterol, and serum albumin.

eGFR, estimated glomerular filtration rate; PCR, protein-to-creatinine ratio.

### Study outcomes

The study endpoint was ESRD and a doubling of the baseline serum creatinine (D-SCr). ESRD was defined as initiation of renal replacement therapy including permanent hemodialysis, peritoneal dialysis, or renal transplantation. We also evaluated the decline rate of eGFR between patients with hypoC3 and patients with normal C3 levels.

**Figure 9:**
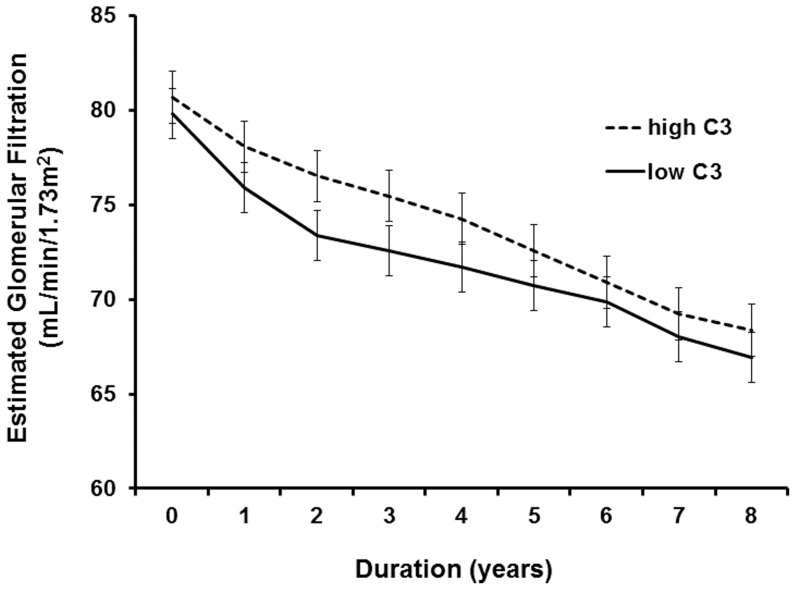
The decline rate of eGFR. Error bars indicate standard error.

### Statistical analysis

Statistical analysis was performed using SPSS version 17.0 (SPSS Inc., Chicago, Illinois, USA). Continuous data were expressed as mean ± SD, and categorical data were expressed as a number (percentage). The two groups were compared using the *t*-test or chi-squared test. The Kolmogorov-Smirnov test was used to analyze the normality of the distribution of parameters. Nonparametric variables were expressed as median and interquartile range and compared using the Mann–Whitney test or Kruskal–Wallis test. Probability of renal survival curves were generated by the Kaplan-Meier method, and between-group survival was compared by the log-rank test. The independent prognostic values of clinical and pathological parameters for the study outcomes were analyzed by multiple Cox regression analyses. Hazard ratios (HRs) and 95% confidence intervals (CIs) were calculated with the use of the estimated regression coefficients and standard errors in the Cox regression analysis. The predictive value for renal outcome was also analyzed by receiver operating characteristic (ROC) curve analysis with calculated area under the ROC curve (AUC). Finally, to compare the decline rate of eGFR between the two groups, multivariate linear regression analysis was conducted. All probabilities were two-tailed and the level of significance was set at 0.05.

## Results

### Baseline characteristics

The demographic, clinical, and biochemical characteristics of the study population are shown in Table 1. Of the 343 patients, 66 patients (19.2%) had C3 levels below the lower limit of the normal range (<90 mg/dl) and were considered to have hypoC3. A scattered plot of each level of serum C3 was presented in Figure 3. Compared to patients with C3 ≥90 mg/dl, those with C3 <90 mg/dl were younger (31.1±9.2 vs. 35.4±12.1 years, P = 0.002) and had lower mean arterial blood pressure (91.4±9.5 vs. 94.8±11.7 mmHg, P = 0.031). Patients with uncontrolled hypertension were more common in patients with C3 ≥90 mg/dl. Of note, these patients had higher BMI, and cholesterol and triglyceride levels than patients with C3 levels <90 mg/dl (Table 1). Between the two groups, however there were no differences in eGFR, UPCR, Ig levels, CRP, comorbidities, or medications including immuno-suppressants and antihypertensive agents during follow-up. Meanwhile, no patients underwent tonsillectomy for the prevention of disease progression in our study.

In patients with nephrotic syndrome, serum C3 levels were 97.8±14.7 mg/dl and 6 (15.8%), 25 (65.7%), and 7 (18.4%) patients had mesangial C3 deposits of 0, 1+, and 2+ or more, respectively. Whether complement activation is involved in the development of nephrotic syndrome in IgA nephropathy is currently unknown. We recently demonstrated that IgAN patients with nephrotic syndrome exhibited different features and had worse prognosis compared with patients with typical IgA nephropathy [19]. In addition, they are usually more likely to be treated with immunosuppressive drugs. These may lead to a biased result, thus patients with nephrotic syndrome were excluded from the analysis.

### Pathologic findings

The histopathologic features from renal biopsies between patients with and without hypoC3 are presented in Table 2. Patients with hypoC3 had higher grade mesangial deposition of C3 (2+ to 3+) than those without hypoC3 (28.8 vs. 18.8%, P = 0.011). There were no differences in mesangial hypercellularity, glomerulosclerosis, endocapillary hypercellularity, tubulointerstitial lesions, arteriosclerosis, and mesangial deposition of IgG or IgA between the two groups (Table 2). The histopathologic features were further compared according to mesangial C3 deposition. Mesangial hypercellularity (C3 deposition 0, 9.4%; 1+, 29.7%; 2+∼3+, 49.3%; P<0.001) and high-grade tubular atrophy/interstitial fibrosis (C3 deposition 0, 7.5%; 1+, 10.5%; 2+∼3+, 14.1%; P<0.001) were more prominent as the mesangial area of C3 deposition increased (Figure 4). However, there was no significant difference in segmental glomerulosclerosis or endocapillary hypercellularity. On the other hand, serum C3 levels decreased significantly from 0 to 2+∼3+ mesangial C3 deposition (0, 111.7±18.0; 1+, 104.3±17.1; 2+∼3+, 98.6±14.2 mg/dl; P<0.001) (Figure 5).

### Renal Outcomes

During a mean follow-up of 53.7±30.1 months, 14 (4.1%) developed ESRD and 29 patients (8.5%) reached the end point of doubling of the baseline serum creatinine levels. There was no patient who progressed to ESRD before reaching doubling of the baseline serum creatinine. ESRD occurred in 5 patients (7.6%) with hypoC3 compared with 9 patients (3.2%) with normal C3 levels (P = 0.11, Table 3). However, 12 patients (18.2%) with hypoC3 reached D-SCr compared with 17 patients (6.1%) with normal C3 levels (HR, 3.59; 95% CI, 1.56–8.28; P = 0.003) (Table 3 and Table 4, Model 1) and a 10-year renal survival rate was significantly lower in the former (Figure 6A, P = 0.006). In addition, the expanded mesangial deposition of C3 was associated with worse renal survival (Figure 7B). ESRD occurred in 1 (1.9%), 9 (4.1%), and 4 (5.6%) patients with mesangial C3 deposition of 0, 1+, and 2+ to 3+, respectively (P = 0.30, Table 3). However, the risk of reaching D-SCr was significantly higher in patients with mesangial C3 deposition 2+ to 3+ than in patients without deposition (HR 14.24; 95% CI, 2.03–99.87; P = 0.008) (Table 4, Model 2). In a multivariable model in which serum C3 levels were treated as a continuous variable, hypoC3 significantly predicted renal outcome of D-SCr (per 1 mg/dl increase of C3; HR, 0.96; 95% CI, 0.93–0.98; P = 0.002) (Table 4, Model 1). When both serum C3 levels and mesangial C3 deposition were included in a multivariate model, these two parameters remained independent predictors of adverse renal outcomes (Table 4, Model 3 and 4). In ROC curve analysis, serum C3 levels had a significant predictive value for the renal outcome (AUC = 0.642, P = 0.011), although the predictive value of serum C3 was lower than UPCR (AUC = 0.819, P<0.001) or eGFR (AUC = 0.781, P<0.001) (Figure 6). On the other hand, patients with mesangial hypercellularity, segmental glomerulosclerosis, endocapillary proliferation, and tubular atrophy/interstitial fibrosis had significantly lower renal survival than those without such findings (P<0.05) (Figure 8).

In the multivariate linear regression analysis adjusted for clinical, laboratory, and histologic factor, the decline rate of eGFR was greater in patients with hypoC3 than in patients with normal C3 levels only up to 4 years after the diagnosis. However, overall decline rate of eGFR did not differ between the two groups (Figure 9).

## Discussion

This study showed that both decreased circulating C3 levels and mesangial C3 deposition were associated with deterioration of kidney function in patients with IgAN independent of heavy proteinuria and other unfavorable histopathologic features such as glomerular sclerosis or interstitial fibrosis. Our findings suggest that decreased serum C3 levels and C3 deposition within the mesangium may provide prognostic value in these patients.

In patients with IgAN, O-linked carbohydrates in the hinge region of IgA1 molecule are under-galactosylated and defective IgA1 forms circulating or *in situ* immune complexes [20]. Subsequent deposition of immune complexes within the mesangium plays a key role in the pathogenesis of IgAN. Interestingly, mesangial C3 deposits are often observed along with IgA, suggesting that complement activation may also be involved in pathogenesis. In fact, many studies have previously suggested that local complement system in the glomeruli is activated via the alternative or the mannose-binding lectin (MBL) pathway in IgAN [7]–[12]. Although there is a general agreement that C3 deposits are caused predominantly by complement activation via the alternative pathway in IgAN, several studies have recently suggested that the lectin pathway of complement may also be involved in the progression of disease [10]–[12]. In particular, Roos et al. reported that activation of the lectin pathway was associated with more severe renal damage in IgAN [11]. In their study, complement activation occurred via the alternative pathway in 75% of patients whereas glomerular deposition of MBL, L-ficolin, and C4d, which was indicative of activation of complement via the lectin pathway, was observed in 25% of patients [11]. However, it is uncertain whether such complement activation may affect the long-term outcomes in patients with IgAN. Komatsu et al. showed that C3 deposition within the mesangium significantly correlated with severe histologic lesions using kidney specimens from patients with IgAN [21]. In line with their findings, in the present study, we clearly showed that patients with higher grade mesangial deposition of C3 had worse histologic findings such as mesangial hypercellularity and tubular atrophy/interstitial fibrosis than those with lower grade deposition. Because such histologic features are apparently associated with worse prognosis [22], [23], it can be presumed that patients with mesangial C3 deposition have worse renal outcomes compared with those without deposition as seen in our study. Taken together, it can be suggested that complement activation may mediate further renal injury and that mesangial C3 deposition may have prognostic value in IgAN.

Although previous studies demonstrated that complement activation occurred locally in IgAN, several studies reported that systemic complement activation may also be present [13], [14]. In particular, Zwirner et al. showed that activated C3 levels in the plasma were elevated in patients with IgAN and correlated with deterioration in renal function [14], suggesting that systemic complement activation may also be involved in the progression of IgAN. However, measurement of activated C3 is not widely available because slow *ex vivo* generation of activated C3 can be observed even during storage at −70°C [24]. Consumption of complement factors is reflected by either decreased levels of individual proteins such as C3 and C4 or depressed total complement hemolytic activity (CH50), as well as the production of complement activation split products [25]. In this regard, decreased serum C3 levels in our 66 patients are possibly due to C3 consumption due to systemic complement activation although other components of complement cascade and C3 splits were not measured. To date, clinical implication of decreased serum C3 levels in patients with IgAN has not yet been explored. Several Japanese studies have examined the clinical utility of C3. Tomino et al. showed that serum IgA/C3 ratio might be of help in diagnosing IgAN [26]. In addition, renal survival was significantly decreased in patients with a higher serum IgA/C3 ratio than those with a lower ratio [21]. Interestingly, in a study by Komatsu et al., serum C3 levels were decreased in patients with IgAN with severe histologic lesions compared with those seen in non-IgAN. Unfortunately, they did not assess the prognostic value of decreased serum C3 levels. In the present study, we showed for the first time that patients with decreased serum C3 levels had worse renal survival than those with higher C3 levels. Moreover, decreased serum C3 levels were independently predictive of renal outcome in the multivariate analysis even after adjustment for factors known to be associated with worse prognosis such as proteinuria and decreased renal function at presentation although predictive value of low C3 levels were not so potent as these conventional factors. It should be noted that many physicians are not aware of the significance of decreased serum C3 levels in patients with IgAN although hypoC3 is not common. In this regard, our findings deserve particular attention because decreased serum C3 levels may be another useful biomarker to predict progression of IgAN.

In contrast to findings by Tomino et al, in this study, IgA/C3 ratio had a trend toward poor renal outcome, but it did not reach statistical significance (data not shown). This can be explained by the fact that simply elevated IgA levels cannot reflect disease activity. In fact, although circulating IgA levels are elevated in patients with IgAN, correlation of the elevated IgA levels with clinical features of the disease is inconsistent [27], [28]. Interestingly, 40 to 50% of first degree relatives of IgAN patients have elevated IgA levels, but most of these persons do not exhibit clinical sings of renal injury [29], [30]. These findings suggest that additional pathogenic factors such as generation of antibodies against IgA or immune-complex formation are required to activate the disease.

It is uncertain whether systemic and local activation of complement system coordinate together or if they exert independent effects although both types of activation have been previously reported in IgAN [7]–[14]. Activation of the complement system was common in patients with systemic lupus erythematosus (SLE), leading to hypocomplementemia and deposition of complement component at sites of tissue injury, particularly in the glomeruli and the skin [31]. This finding suggests that complement activation likely has a role in tissue damage in SLE. In addition, some studies reported that the lectin pathway of the complement system is activated in rheumatoid arthritis. Although levels of lectin pathway proteins were higher in plasma than synovial fluid, paired plasma and synovial fluid levels correlated significantly in all cases [32]. Consistent with these findings, our study showed that serum C3 levels correlated with mesangial C3 deposition, suggesting a possible link between the two complement systems. Despite such a correlation, it is possible that both factors may be independently associated with poor renal survival because there was no significant interaction on multivariable Cox regression analysis (data not shown). However, due to the observational nature of the study, it is difficult to clarify how systemic complement activation is involved in local activation.

Interestingly, in this study, patients with C3 ≥90 mg/dl had higher BMI, blood pressure, and cholesterol levels. Recent previous studies showed that increased complement levels were related to extreme adiposity and insulin resistance [33], [34]. On the contrary, a few studies suggested complement activation in patients with very low BMI such as anorexia nervosa [35]–[37]. All these patients exhibited decreased C3 levels, which might reflect severe comorbid conditions such as malnutrition. However, in this study, it is uncertain whether C3 levels were affected by obesity or nutritional status. Mean BMI of the study subjects were 22.7 kg/m^2^ and there were only 12 patients with BMI>30 kg/m^2^. Even patients with C3 ≥90 mg/dl had a mean BMI of 23.2 kg/m^2^, suggesting that they were not obese. In addition, serum albumin level, which is a good indicator of nutritional status, did not differ between patients with hypoC3 and patients with C3 ≥90 mg/dl. Nevertheless, to examine possible effects of these factors on outcome, we conducted a Cox regression model further adjusted for BMI, cholesterol, and serum albumin and found that decreased C3 levels and mesangial C3 deposits remained significant predictors of renal outcome (Table 4, Model 4).

Not surprisingly, we confirmed that unfavorable histologic features such as mesangial hypercellularity, glomerulosclerosis, and tubular atrophy/interstitial fibrosis, which were previously reported to be poor prognostic factors, were significantly associated with adverse renal outcomes. However, it is unclear how glomerulopathy associated with mesangial IgA deposition leads to tubulointerstitial injury although tubulointerstitial lesions are common in IgAN [38]. It is possible that glomerulotubular cross-talk with mediators such as cytokines, complement, and angiotensin II may contribute to the pathogenesis of tubulointerstitial damage in IgAN as suggested by Chan et al. [39]. Interestingly, in the present study, the severity of tubulointerstitial lesions was associated with the expansion of mesangial C3 deposition. This finding suggests that complement activation may aggravate glomerular injury, eventually resulting in the development of tubulointerstitial damage, and that the complement system may act as a mediator in glomerulotubular cross-talk.

Recent studies showed that other pathologic features such as wide areas of electron dense deposits [40] and thrombotic microangiopathy [41] were associated with adverse outcome in IgAN. Detailed analysis on relationship between mesangial C3 deposition and these pathologic features is beyond the scope of this study. However, we found that there was no correlation between intensity of C3 deposits and areas of electron dense deposits although electron dense deposits were observed in other areas besides paramesangial area. It is possible that complement activation in response to immune complex may differ depending on mesangial or paramesangial areas. In addition, thrombotic microangiopathy was found in only 25 (7.3%) patients, which is quite low compared with 53% in a study by Karoui et al. This is probably because our study subjects were relatively young and blood pressure was well controlled. In fact, in a study by Karoui et al, 71% had uncontrolled hypertension, which was associated with thrombotic microangiopathy. In contrast, only 38 (11.1%) patients in our study had systolic blood pressure >140 mmHg and intensity C3 deposits were not related with thrombotic microangiopathy. Furthermore, Karoui et al. found no genetic mutation of complement factor H and I in patients with severe thrombotic microangiopathy, suggesting the alternative pathway is less likely involved in this lesion. Whether complement activation can contribute to more depositions of immune-complex besides paramesangial area or thrombotic microangiopathy requires further in-depth investigations.

### Limitations

Our study had several limitations. First, this was a retrospective study, thus the observational nature of the present study limits our findings suggesting that complement activation actually contributes to the progression of IgAN. Second, other complement components including activated C3, C4-C3 complexes, or soluble C5b-9 were not available. We also did not perform the additional staining for MBL, C4d, and C3c in glomeruli, which were previously suggested to be possible predictors of disease activity as alternatives to C3 [9], [12]. Third, serum C3 levels were only measured at the time of renal biopsy. Thus, whether hypoC3 persisted throughout the disease course is unknown. Interestingly, there were five patients who had follow-up data for serum C3 levels with a median duration of 22 months. They had persistently decreased serum C3 levels and four of them reached the doubling of the baseline serum creatinine. Therefore, it would be helpful to monitor the level serially to further clarify the clinical implications of hypoC3 in IgAN. Fourth, serum C3 levels and mesangial C3 deposition were not associated with the development of ESRD. This finding is partly due to the fact that ESRD occurred in only 14 patients (4.2%) during the follow-up period, thus resulting in a lack of statistical power. In addition, overall decline rate of eGFR did not differ between patients with hypoC3 and patients with normal C3 levels. It should be noted that most patients with hypoC3 reached the endpoints within 4 years after the baseline evaluation. This can explain the faster decline in eGFR in these patients until 4 years. However, it is possible that small number of events did not have adequate statistical power to see the difference in eGFR decline. Fifth, the presence of other diseases exhibiting both hypoC3 and mesangial IgA deposition could not entirely be excluded. However, we conducted a thorough pathologic examination and excluded patients with conditions such as systemic lupus erythematosus and IgA-dominant acute post-infectious glomerulonephritis. Moreover, we confirmed that autoantibodies such as antinuclear antibody or anti-DNA antibody were negative in all patients with hypoC3. Sixth, intensity of immunofluorescence may not be correct because of different condition of immunofluorescent staining, storage time, or altered antigenicity of immune complex by environmental proteases. However, in our institute, immunofluorescence pictures were generally taken immediately after biopsy samples were processed. Furthermore, to quantify the immunofluorescence intensity, these pictures were converted to digital images and analyzed using ImageJ software. Seventh, serum C3 levels were mildly decreased in 66 patients with hypoC3, suggesting that the disease may not truly be a ‘flare-up’, which can be seen in severe lupus nephritis [42]. It is unknown whether such a mild decrease in C3 levels may affect the clinical outcomes. Considering the fact that IgAN exhibits a slowly progressive course, it is possible that indolent inflammatory process is still underway even in such a mild hypoC3status. Based on our finding that renal survival rate was lower in patients with hypoC3 compared to those with normal C3 levels although no significant difference in baseline histopathologic features was observed, we surmise that complement activation may contribute to the development of slowly progressive renal injury for an extended period of time. Finally, clinical significance of C3 levels was reported mostly from studies involving Asian population [21], [26]. In addition, a prior study suggested a geographical variability in long-term outcomes of IgA nephropathy [43]. Therefore, our results may not be extrapolated to other ethnic populations.

### Conclusion

This study showed that both decreased circulating C3 levels and mesangial C3 deposition were independently associated with poor renal outcome in patients with IgAN. These findings suggest that systemic and local activation of complement may play a role in the progression of IgAN and decreased serum C3 levels and mesangial C3 deposition may have prognostic value in the management of these patients.

## References

[1] D'Amico G (1987). The commonest glomerulonephritis in the world: IgA nephropathy.. Q J Med.

[2] D'Amico G, Colasanti G, Barbiano di Belgioioso G, Fellin G, Ragni A (1987). Long-term follow-up of IgA mesangial nephropathy: clinico-histological study in 374 patients.. Semin Nephrol.

[3] Li PK, Ho KK, Szeto CC, Yu L, Lai FM (2002). Prognostic indicators of IgA nephropathy in the Chinese–clinical and pathological perspectives.. Nephrol Dial Transplant.

[4] D'Amico G (2004). Natural history of idiopathic IgA nephropathy and factors predictive of disease outcome.. Semin Nephrol.

[5] Walsh M, Sar A, Lee D, Yilmaz S, Benediktsson H (2010). Histopathologic features aid in predicting risk for progression of IgA nephropathy.. Clin J Am Soc Nephrol.

[6] Oortwijn BD, Eijgenraam JW, Rastaldi MP, Roos A, Daha MR (2008). The role of secretory IgA and complement in IgA nephropathy.. Semin Nephrol.

[7] Hiemstra PS, Gorter A, Stuurman ME, Van Es LA, Daha MR (1987). Activation of the alternative pathway of complement by human serum IgA.. Eur J Immunol.

[8] Stad RK, Bruijn JA, van Gijlswijk-Janssen DJ, van Es LA, Daha MR (1993). An acute model for IgA-mediated glomerular inflammation in rats induced by monoclonal polymeric rat IgA antibodies.. Clin Exp Immunol.

[9] Nakagawa H, Suzuki S, Haneda M, Gejyo F, Kikkawa R (2000). Significance of glomerular deposition of C3c and C3d in IgA nephropathy.. Am J Nephrol.

[10] Endo M, Ohi H, Ohsawa I, Fujita T, Matsushita M (1998). Glomerular deposition of mannose-binding lectin (MBL) indicates a novel mechanism of complement activation in IgA nephropathy.. Nephrol Dial Transplant.

[11] Roos A, Rastaldi MP, Calvaresi N, Oortwijn BD, Schlagwein N (2006). Glomerular activation of the lectin pathway of complement in IgA nephropathy is associated with more severe renal disease.. J Am Soc Nephrol.

[12] Espinosa M, Ortega R, Gomez-Carrasco JM, Lopez-Rubio F, Lopez-Andreu M (2009). Mesangial C4d deposition: a new prognostic factor in IgA nephropathy.. Nephrol Dial Transplant.

[13] Wyatt RJ, Kanayama Y, Julian BA, Negoro N, Sugimoto S (1987). Complement activation in IgA nephropathy.. Kidney Int.

[14] Zwirner J, Burg M, Schulze M, Brunkhorst R, Gotze O (1997). Activated complement C3: a potentially novel predictor of progressive IgA nephropathy.. Kidney Int.

[15] Nasr SH, D'Agati VD (2011). IgA-dominant postinfectious glomerulonephritis: a new twist on an old disease.. Nephron Clin Pract 119: c18–25; discussion c26.

[16] Giannakakis K, Faraggiana T (2011). Histopathology of lupus nephritis.. Clin Rev Allergy Immunol.

[17] Levey AS, Bosch JP, Lewis JB, Greene T, Rogers N (1999). A more accurate method to estimate glomerular filtration rate from serum creatinine: a new prediction equation. Modification of Diet in Renal Disease Study Group.. Ann Intern Med.

[18] Cattran DC, Coppo R, Cook HT, Feehally J, Roberts IS (2009). The Oxford classification of IgA nephropathy: rationale, clinicopathological correlations, and classification.. Kidney Int.

[19] Kim JK, Kim JH, Lee SC, Kang EW, Chang TI (2012). Clinical features and outcomes of IgA nephropathy with nephrotic syndrome.. Clin J Am Soc Nephrol.

[20] Floege J, Feehally J (2000). IgA nephropathy: recent developments.. J Am Soc Nephrol.

[21] Komatsu H, Fujimoto S, Hara S, Sato Y, Yamada K (2004). Relationship between serum IgA/C3 ratio and progression of IgA nephropathy.. Intern Med.

[22] Radford MG, Donadio JV, Bergstralh EJ, Grande JP (1997). Predicting renal outcome in IgA nephropathy.. J Am Soc Nephrol.

[23] Myllymaki JM, Honkanen TT, Syrjanen JT, Helin HJ, Rantala IS (2007). Severity of tubulointerstitial inflammation and prognosis in immunoglobulin A nephropathy.. Kidney Int.

[24] Janssen U, Bahlmann F, Kohl J, Zwirner J, Haubitz M (2000). Activation of the acute phase response and complement C3 in patients with IgA nephropathy.. Am J Kidney Dis.

[25] Tsokos GC (2004). Exploring complement activation to develop biomarkers for systemic lupus erythematosus.. Arthritis Rheum.

[26] Tomino Y, Suzuki S, Imai H, Saito T, Kawamura T (2000). Measurement of serum IgA and C3 may predict the diagnosis of patients with IgA nephropathy prior to renal biopsy.. J Clin Lab Anal.

[27] van der Boog PJ, van Kooten C, van Seggelen A, Mallat M, Klar-Mohamad N (2004). An increased polymeric IgA level is not a prognostic marker for progressive IgA nephropathy.. Nephrol Dial Transplant.

[28] Feehally J, Beattie TJ, Brenchley PE, Coupes BM, Mallick NP (1986). Sequential study of the IgA system in relapsing IgA nephropathy.. Kidney Int.

[29] Gharavi AG, Moldoveanu Z, Wyatt RJ, Barker CV, Woodford SY (2008). Aberrant IgA1 glycosylation is inherited in familial and sporadic IgA nephropathy.. J Am Soc Nephrol.

[30] Suzuki H, Kiryluk K, Novak J, Moldoveanu Z, Herr AB (2011). The pathophysiology of IgA nephropathy.. J Am Soc Nephrol.

[31] Cook HT, Botto M (2006). Mechanisms of Disease: the complement system and the pathogenesis of systemic lupus erythematosus.. Nat Clin Pract Rheumatol.

[32] Ammitzboll CG, Thiel S, Ellingsen T, Deleuran B, Jorgensen A (2011). Levels of lectin pathway proteins in plasma and synovial fluid of rheumatoid arthritis and osteoarthritis.. Rheumatol Int.

[33] Ohsawa I, Inoshita H, Ishii M, Kusaba G, Sato N (2010). Metabolic impact on serum levels of complement component 3 in Japanese patients.. J Clin Lab Anal.

[34] Hernandez-Mijares A, Jarabo-Bueno MM, Lopez-Ruiz A, Sola-Izquierdo E, Morillas-Arino C (2007). Levels of C3 in patients with severe, morbid and extreme obesity: its relationship to insulin resistance and different cardiovascular risk factors.. Int J Obes (Lond).

[35] Palmblad J, Fohlin L, Norberg R (1979). Plasma levels of complement factors 3 and 4, orosomucoid and opsonic functions in anorexia nervosa.. Acta Paediatr Scand.

[36] Sigal LH, Snyder BK (1989). Low serum complement levels in anorexia nervosa.. Am J Dis Child.

[37] Flierl MA, Gaudiani JL, Sabel AL, Long CS, Stahel PF (2011). Complement C3 serum levels in anorexia nervosa: a potential biomarker for the severity of disease?. Ann Gen Psychiatry.

[38] Roufosse CA, Cook HT (2009). Pathological predictors of prognosis in immunoglobulin A nephropathy: a review.. Curr Opin Nephrol Hypertens.

[39] Chan LY, Leung JC, Lai KN (2004). Novel mechanisms of tubulointerstitial injury in IgA nephropathy: a new therapeutic paradigm in the prevention of progressive renal failure.. Clin Exp Nephrol.

[40] Kusaba G, Ohsawa I, Ishii M, Inoshita H, Takagi M (2012). Significance of broad distribution of electron-dense deposits in patients with IgA nephropathy.. Med Mol Morphol.

[41] El Karoui K, Hill GS, Karras A, Jacquot C, Moulonguet L (2012). A clinicopathologic study of thrombotic microangiopathy in IgA nephropathy.. J Am Soc Nephrol.

[42] Birmingham DJ, Irshaid F, Nagaraja HN, Zou X, Tsao BP (2010). The complex nature of serum C3 and C4 as biomarkers of lupus renal flare.. Lupus.

[43] Geddes CC, Rauta V, Gronhagen-Riska C, Bartosik LP, Jardine AG (2003). A tricontinental view of IgA nephropathy.. Nephrol Dial Transplant.

